# Gait analysis of a patient after femoral nerve and malignant soft tissue tumor resections: a case report

**DOI:** 10.1186/s12891-024-07258-8

**Published:** 2024-02-12

**Authors:** Yuta Kubota, Yuta Tsubouchi, Masaya Anan, Masanori Kawano, Tatsuya Iwasaki, Ichiro Itonaga, Shinichi Ikeda, Masashi Kataoka, Hiroshi Tsumura, Nobuhiro Kaku, Kazuhiro Tanaka

**Affiliations:** 1 Department of Orthopaedic Surgery, Faculty of Medicine, Oita University, 1-1 Idaigaoka Hasama, Yufu City, Oita, 879-5593 Japan; 2 School of Physical Therapy, Faculty of Rehabilitation, Reiwa Health Sciences University, 2-1-12 Wajirogaoka, Higashi-Ku, Fukuoka, 811-0213 Japan; 3 Physical Therapy Course of Study, Faculty of Welfare and Health Sciences, Oita University, 700 Dannoharu, Oita-City, Oita, 870-1192 Japan; 4 Department of Rehabilitation Medicine, Beppu Rehabilitation Center, 1026-10 Tsurumi, Beppu-Shi, Oita, 874-8611 Japan; 5 Department of Advanced Medical Sciences, Faculty of Medicine, Oita University, 1-1 Idaigaoka Hasama, Yufu City, Oita, 879-5593 Japan

**Keywords:** Gait analysis, Femoral nerve resection, Malignant soft tissue tumor, Case report

## Abstract

**Background:**

Malignant femoral soft tissue tumors are occasionally resected together with the femoral nerves, but this can cause loss of knee extensor muscle activity. To the best of our knowledge, no previous reports have detailed the gait analysis of such cases in combination with electromyography. Herein, we report the gait analysis of a patient who underwent left groin synovial sarcoma and left femoral nerve resection 12 years ago.

**Case presentation:**

We analyzed the gait of a 38-year-old man who was able to walk unaided after the resection of a synovial sarcoma in the left groin together with the ipsilateral femoral nerve. The muscle activities of the affected medial (MH) and lateral hamstrings (LH), and lateral heads of the gastrocnemius (GL) were increased during 50–75% of the stance phase. The hip flexion angle of the affected limb was smaller, and the ankle plantar flexion angle of the affected limb was larger than that of the non-affected limb. This means that in the affected limb, the hip and ankle angles were adjusted to prevent knee collapse, and the MH, LH, and GL muscles contributed in the mid- and late-stance phases. Moreover, we found that the hamstring and gastrocnemius of the affected limb worked together to keep the ipsilateral knee extended in the mid-stance phase and slightly flexed in the late-stance phase.

**Conclusions:**

Patients capable of walking after femoral nerve resection may control their hamstrings and gastrocnemius muscles collaboratively to prevent ipsilateral knee collapse in the mid- and late-stance phases.

## Background

Some motor nerve injuries or neuromuscular diseases cause muscle deterioration, and if the deterioration causes impaired gait, other muscles compensate for walking [1]. The motor branches of the femoral nerve supply the sartorius and quadriceps femoris, causing knee extension and aiding hip flexion, resulting in stability during stance [2]. To date, gait analyses in patients after complete resection of the patella and quadriceps femoris [3], those with hemiparetic stroke [4], and those with knee osteoarthritis [5] and sit-to-stand motion analysis in those with knee osteoarthritis [6] have been reported. There are several methods of gait analyses for patients with femoral nerve dysfunction [2, 7] that do not rely on electromyography (EMG). According to Sutherland et al. [8], although joint moments and powers provide useful information, only the net moment created by all the forces crossing the joint can be determined. Thus, additional information, such as EMG, is needed to determine the contribution of individual muscles [8]. However, no studies have reported gait analysis combined with electromyography in a patient with a malignant femoral tumor who underwent both femoral nerve and tumor resections. Herein, we report the gait analysis combined with electromyography for a patient who underwent left groin synovial sarcoma and left femoral nerve resection 12 years ago.

## Case presentation

**Fig. 1:**
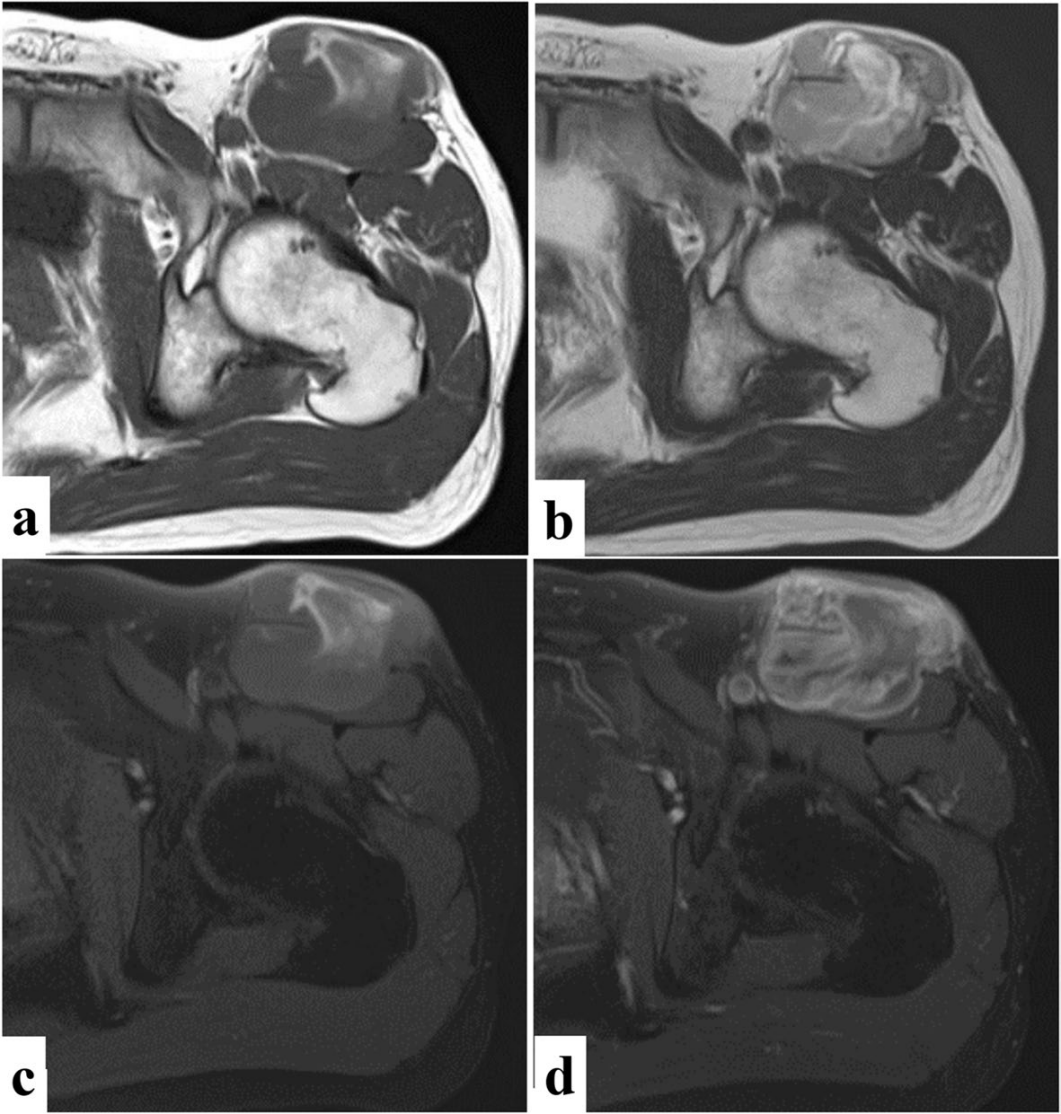
Magnetic resonance images. Magnetic resonance imaging shows a mass lesion in the left femur. The tumor occupying the sartorius muscle has **a** Hypointensity with few hyperintensities on T1-weighted image (WI) and **b** Hyperintensity on T2WI. **c** Fat-saturated T1WI shows some areas of hyperintensity in the mass, and **d** Gadolinium-enhanced fat-saturated T1WI shows enhancement in the mass lesion excluding the central region

A 38-year-old male patient with no significant past medical history presented with a tumor in the left groin. The tumor began to grow and had become painful 2 months prior; the patient visited a neighborhood doctor and was referred to our hospital. Physical examination results revealed that the tumor was 6 cm in diameter, elastic, soft, immobile, and partly tender. However, there was no redness or heat sensation. Radiological examination results revealed opacities without calcification or cortical bone destruction near the tumor. Magnetic resonance imaging (MRI) showed that the tumor occupied the sartorius muscle; measured 65.8 × 62.2 × 50.5 mm; was adjacent to the left femoral neurovascular bundle; and had low overall intensity and partial high intensity on T1-weighted image (WI), moderate-to-high overall intensity with the center exhibiting strong high density on T2WI, some areas of hyperintensity on fat-saturated T1WI, and high intensity on the gadolinium-enhanced fat-saturated T1WI (Fig. 1). Undifferentiated pleomorphic sarcoma, synovial sarcoma, or leiomyosarcoma was suspected.

Histological examination results of the biopsy specimens revealed proliferating, dense, and small spindle cells with oval-to-spindle-shaped nuclei. Immunohistochemistry analysis revealed that these tumor cells were positive for vimentin and MIC2 and negative for CD34, SMA, and S-100P. The *SYT-SSX* fusion gene was detected using reverse-transcription polymerase chain reaction. Therefore, the patient was diagnosed with synovial sarcoma.

**Fig. 2:**
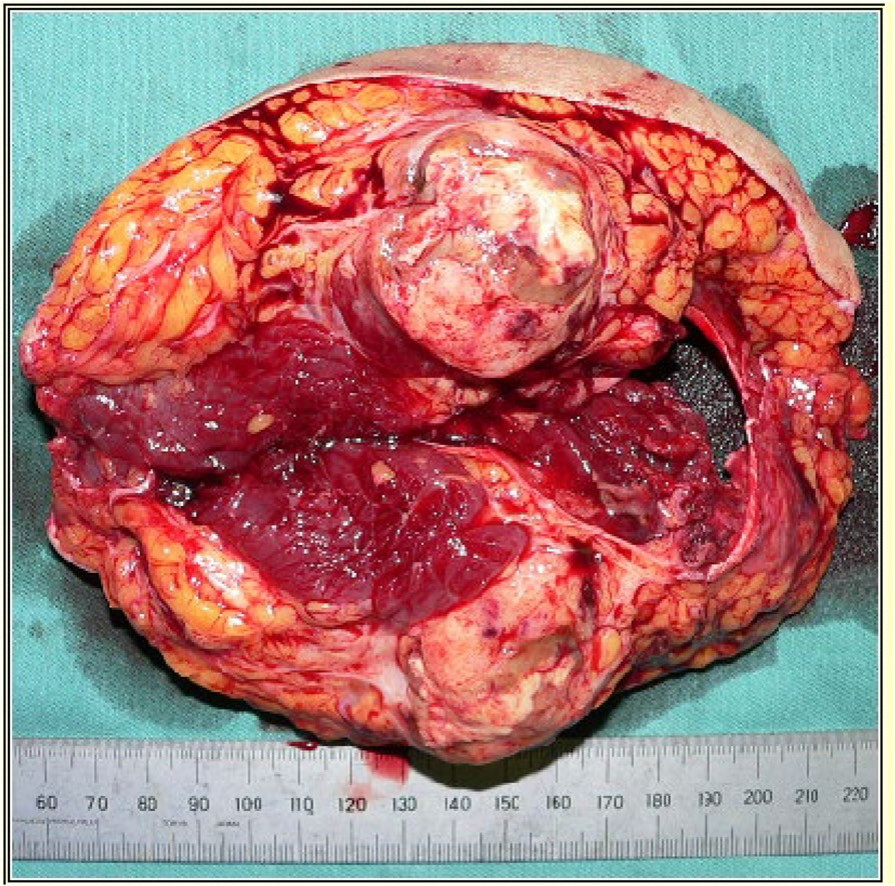
Photographic image of the resected specimen. Photographic image shows the resected specimen

Whole-body contrast-enhanced computed tomography and brain MRI showed no metastatic regions. After administering two-cycle neoadjuvant chemotherapy based on doxorubicin and ifosfamide, wide-margin tumor resection was performed together with the adjacent femoral neurovascular bundle (Fig. 2). The femoral artery and vein were reconstructed with artificial blood vessels, and a rectus abdominis musculocutaneous flap was used for the left groin defect. After the flap was confirmed to be successful, one-cycle adjuvant chemotherapy based on doxorubicin and ifosfamide was administered, followed by conventional radiotherapy consisting of 56 Gy/28 fractions. At discharge, he was able to walk stably with a cane.

Two years later, a subcutaneous tumor was observed in the right temporal region of the head. The tumor was resected and pathologically diagnosed as a metastasis of synovial sarcoma. Post-operative chemotherapy with high-dose ifosfamide was administered. Since then, he has remained disease-free for 12 years.

Gait analysis was performed 12 years postoperatively when the patient was 50 years old. He was able to walk stably without assistance. Motion analysis was performed using a three-dimensional motion analysis system (Nexus, Vicon, Oxford, UK) with 45 infrared-reflecting markers (diameter, 14 mm) on the body, as previously described [9]. Motion capture was conducted using 10 infrared cameras with a sampling rate of 100 frames/sec. Eight ground reaction force plates (AMTI, Watertown, MA, USA) were used at a sampling rate of 1,000 Hz for kinetic data collection. Simultaneously, the muscle activity of the vastus medialis (VM) and lateralis (VL), medial (MH) and lateral hamstrings (LH), and medial (GM) and lateral heads of the gastrocnemius (GL) were collected during the walking test using surface electromyography (Delsys, Natick, MA, USA) with precise electrodes placed according to the surface EMG for the non-invasive assessment of muscles (SENIAM) guidelines [10]. The EMG sampling frequency was 1,000 Hz with a passband of 20–450 Hz. Using maximum voluntary contraction (MVC) activity, we normalized the EMG data from the MH, LH, GM, and GL, excluding those from the VM and VL, as a percentage of MVC values. Due to the very low electrical activity sensed in the left quadriceps femoris, drawing EMG from left VM and VL by %MVC was difficult. Therefore, only the EMGs of VM and VL are shown in volts. A walking test was performed by allowing the patient to walk along a 10-m walkway at a comfortable speed 10 times, and 10 steps from each leg were sampled unilaterally and averaged. A previously reported three-dimensional model [9] was applied. All variables were normalized to 100% of the stance phase duration.

**Fig. 3:**
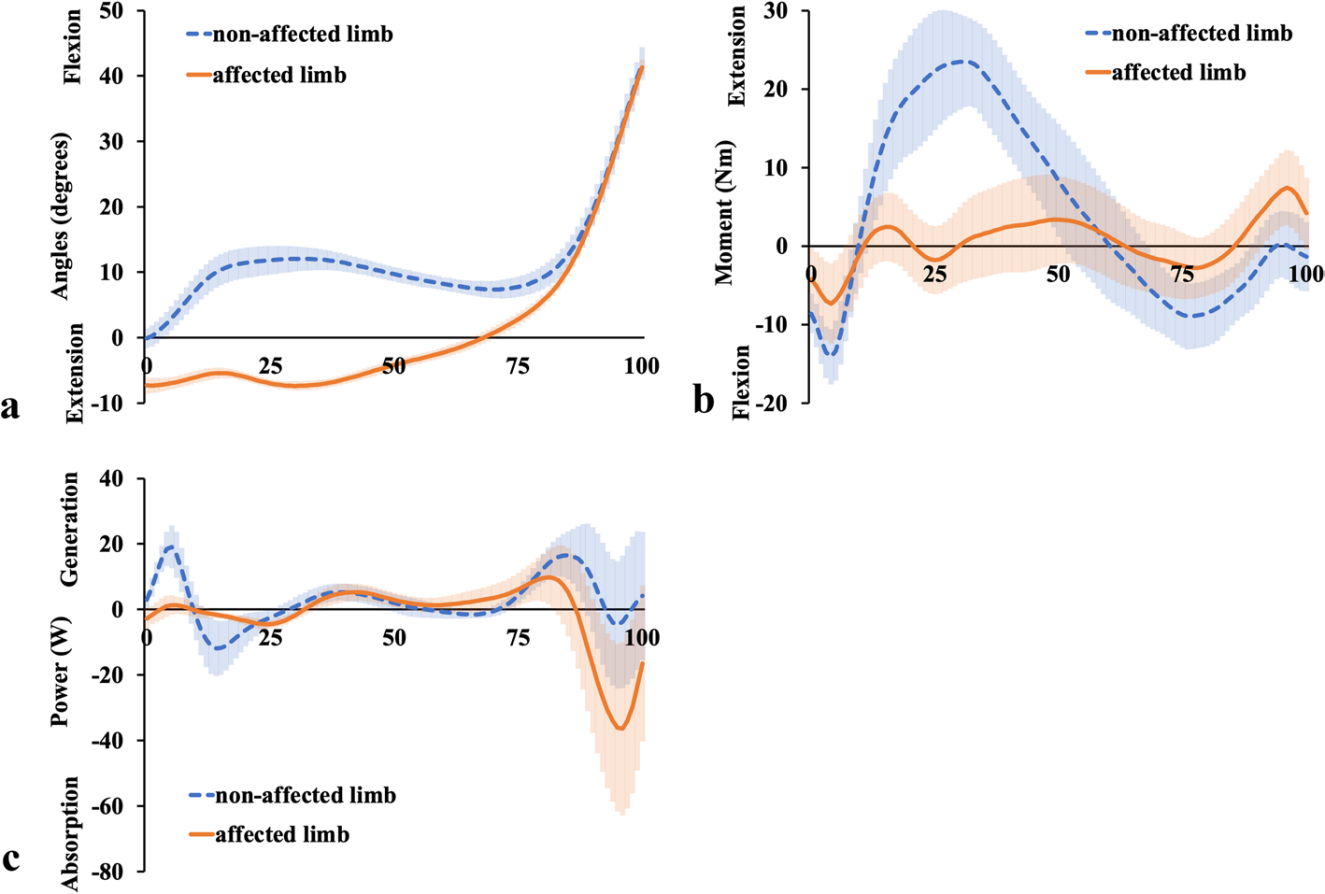
Knee joint kinematic and kinetic results. Knee joint **a** Angular displacements, **b** Moment, and **c** Power are shown. The orange solid lines show the affected limb data, and the blue dashed lines show the non-affected limb data. The shaded areas indicate standard deviation

Normal knee kinematics during walking were observed during the entire stance phase for the non-affected limb (Fig. 3a). Knee flexion started from 0° at initial contact, increased to over 10° at the loading response, and then became slightly extended but flexed slightly at the late-stance phase, leading to the swing phase. Conversely, while walking at a comfortable speed, the knee angle of the affected limb started with slight hyperextension at initial contact, was gradually flexed during the mid-stance phase, and was able to begin toe-off without knee collapse (Fig. 3a). As shown in Fig. 3b, the knee joint moment for the non-affected limb started as flexion at initial contact, became extension from the loading response to the mid-stance phase, became flexion again in the late-stance phase, and then moved towards the swing phase. In contrast, the knee joint moment for the affected limb was generally small, at approximately 0 Nm. The change in knee joint moment in the affected limb from flexion at initial contact to extension in the loading response was the same as that in the non-affected limb. After these phases, the knee extension moment in the affected limb was maintained from the loading response to the mid-stance phase, except for the flexion moment at approximately 25% (Fig. 3b). According to Fig. 3c, the power of the affected limb in the loading response was weaker than that of the non-affected limb, but the power generation towards the extension moment in the mid-stance phase was almost the same in the bilateral limbs.

**Fig. 4:**
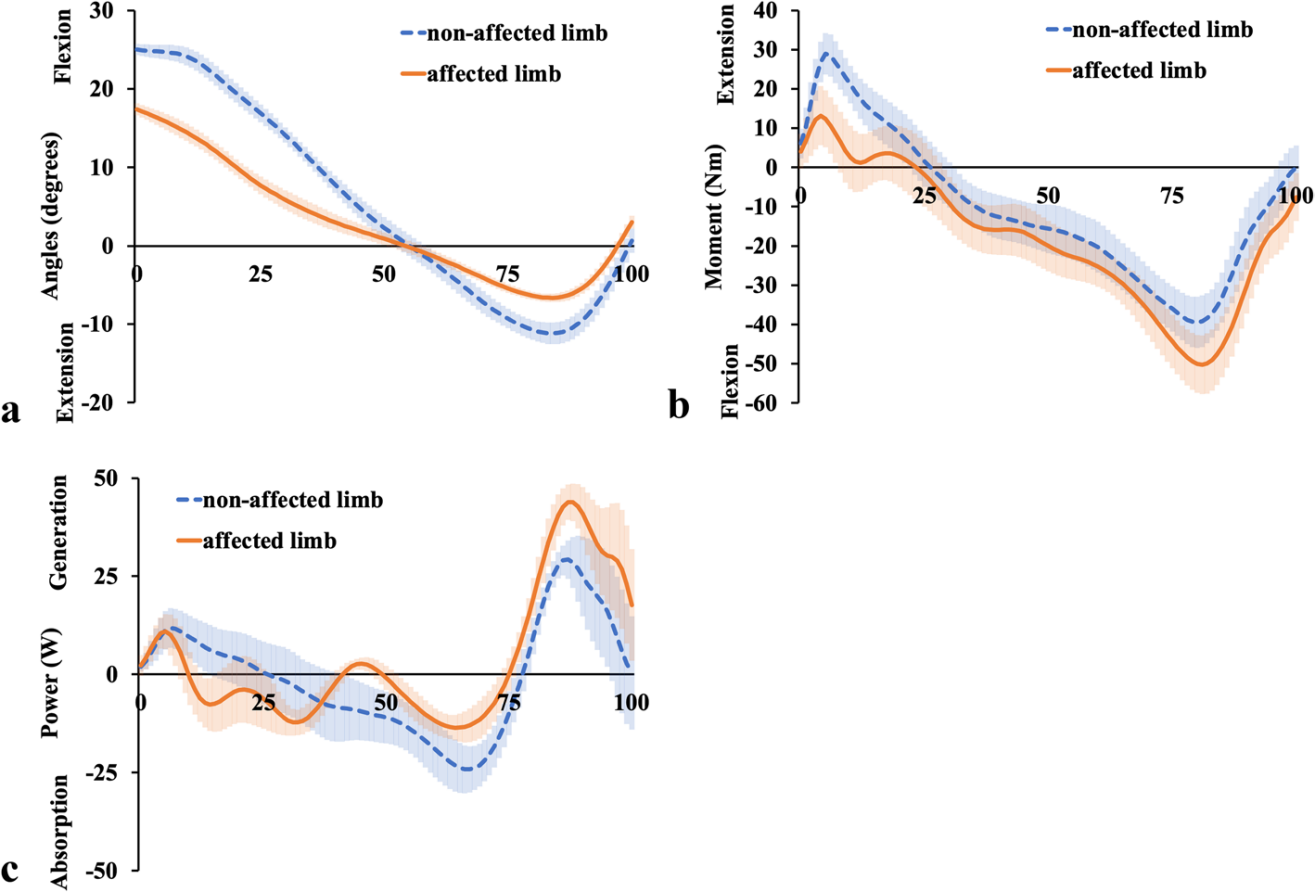
Hip joint kinematic and kinetic results. Hip joint **a** Angular displacements, **b** Moment, and **c** Power are shown. The orange solid lines show the affected limb data, and the blue dashed lines show the non-affected limb data. The shaded areas indicate standard deviation

Next, according to the hip kinematics (Fig. 4a), both hips were flexed during 0–50% of the stance phase, following extension. The hip flexion angle during 0–50% of the stance phase and the extension angle during 50–100% of the stance phase for the affected limb were smaller than that for the non-affected limb. The overall trend in kinetics is shown in Fig. 4b; the extension moment of the affected hip was slightly smaller than that of the non-affected limb. Figure 4c shows that the power of the affected limb fluctuated around zero more frequently than that of the non-affected limb.

**Fig. 5:**
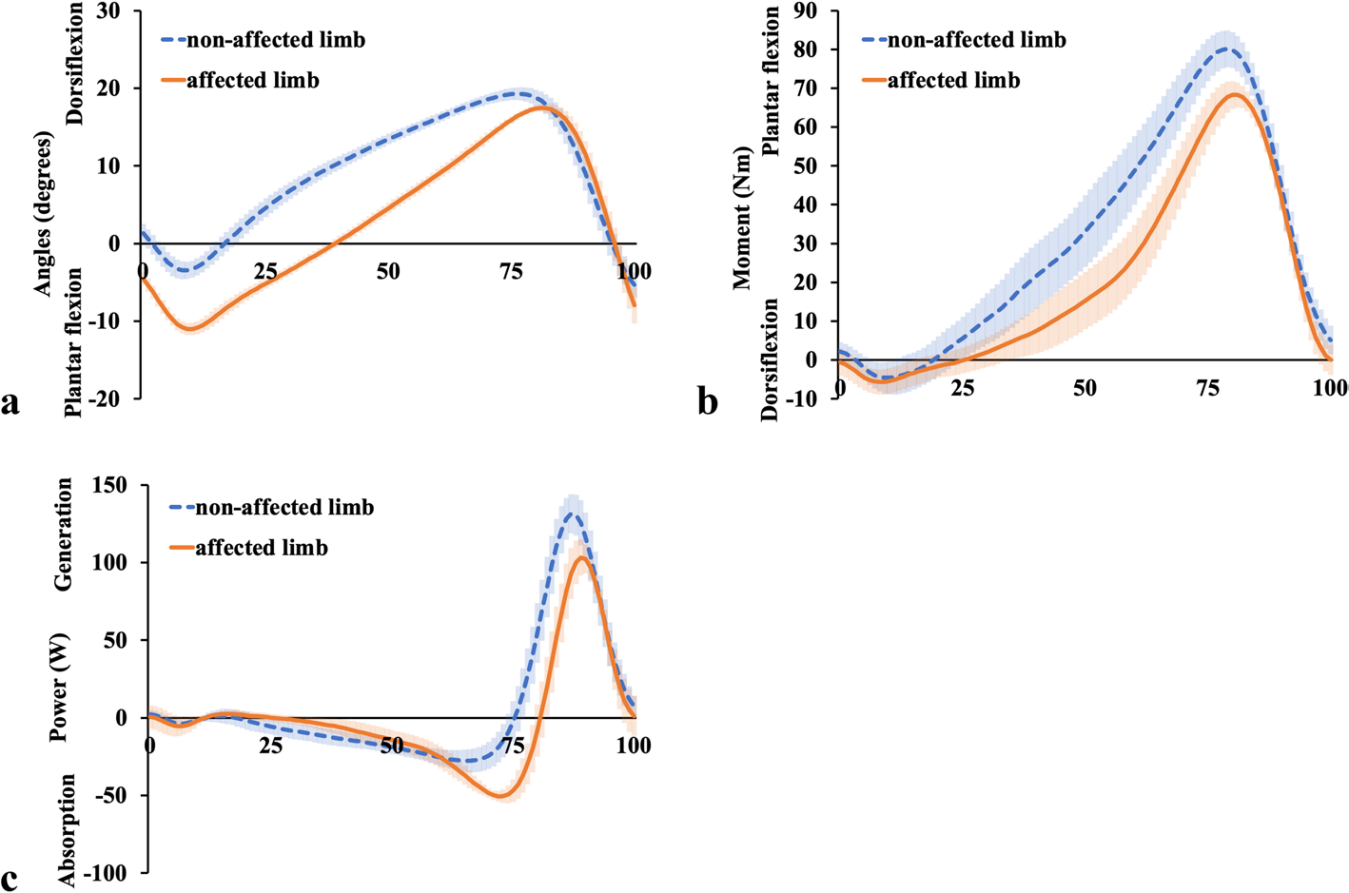
Ankle joint kinematic and kinetic results. Ankle joint **a** Angular displacements, **b** Moment, and **c** Power are shown. The orange solid lines show the affected limb data, and the blue dashed lines show the non-affected limb data. The shaded areas indicate standard deviation

Finally, the ankle plantar flexion angle of the affected limb was larger from the initial contact to the mid-stance phase (Fig. 5a), its moment toward the plantar flexion was smaller during the entire phase (approximately) (Fig. 5b), and its power absorption by the plantar flexor muscles was larger in the late-stance phase (Fig. 5c) than that of the non-affected limb.

**Fig. 6:**
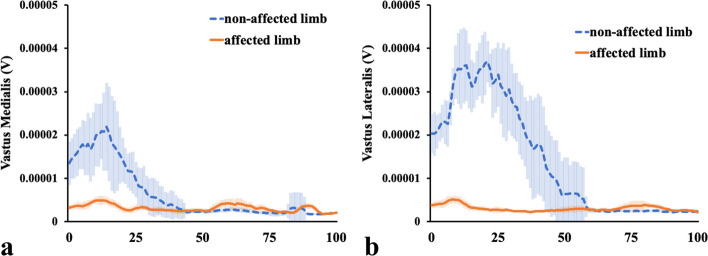
Electromyographic data at the vastus medialis and lateralis. The figures show electromyographic data at the **a** Vastus medialis and **b** Lateralis. The orange solid lines show the affected limb data, and the blue dashed lines represent the non-affected limb data. The shaded areas indicate the standard deviation. V, volts

**Fig. 7:**
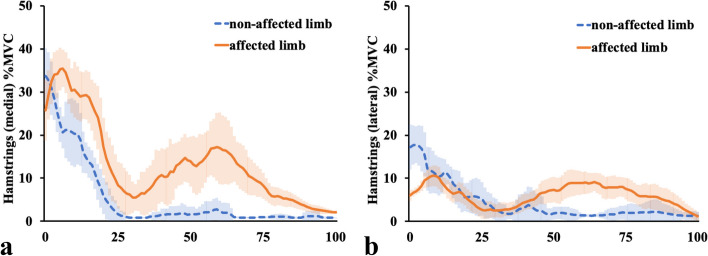
Electromyographic data at the medial and lateral hamstrings. The figures show electromyographic data at the medial (**a**) and lateral (**b**) hamstrings. The orange solid lines show the affected limb data, and the blue dashed lines represent the non-affected limb data. The shaded areas indicate the standard deviation. MVC, maximum voluntary contraction

**Fig. 8:**
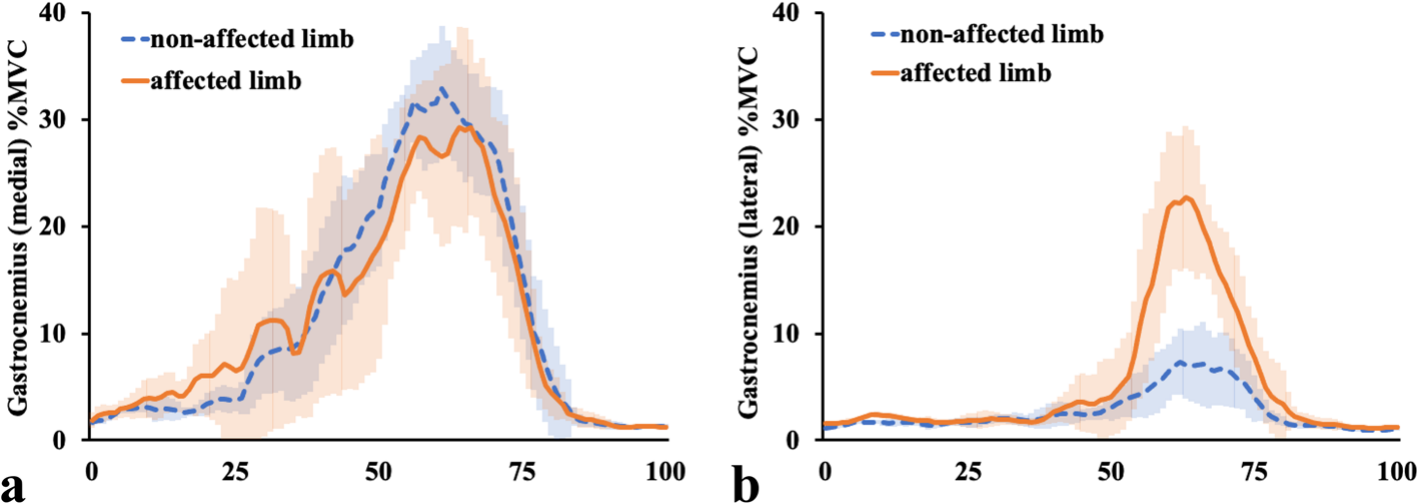
Electromyographic data at the medial and lateral gastrocnemius. The figures show electromyographic data at the medial (**a**) and lateral (**b**) gastrocnemius. The orange solid lines show the affected limb data, and the blue dashed lines represent the non-affected limb data. The shaded areas indicate the standard deviation. MVC, maximum voluntary contraction

It was clear that the EMG for both the VM and VL in the affected limb did not show a valid signal (Fig. 6a, b). EMG showed that the electric activity of both the MH (Fig. 7a) and LH (Fig. 7b) of the affected limb increased from the mid-stance phase to the late-stance phase compared with that of the non-affected limb. The electric activity of both the GM (Fig. 8a) and GL (Fig. 8b) of the bilateral limbs increased from their values at 50% of stance to the late-stance phase, and that of the GL of the affected limb was larger than that of the non-affected limb.

## Discussion and conclusions

This case report describes the gait analysis of a patient with a resected femoral nerve who did not regain any quadriceps femoris contraction but was able to walk without aids.

Lohmann et al. [7] reported that in the affected limb, the hip extension moment and the knee flexor net moments increased in the early stance, and the knee flexor moment and ankle plantar flexor power absorption increased in the later stance, compensating for the quadriceps femoris dysfunction. However, it was unclear whether their cases involved complete loss of the quadriceps femoris contraction because they did not use EMG and their patients probably had intact femoral nerves; therefore, they only inferred that the compensation for quadriceps femoris dysfunction was from the biarticular muscles, such as the hamstrings and gastrocnemius muscles. Two major differences were found between our results and those of Lohmann et al. [7]. Firstly, in our study, the affected limb showed extension moment at the knee joint (Fig. 3b), during approximately 25–63% of the stance phase, as opposed to flexion moment in their study. Secondly, in our study, the affected limb showed flexion moment at the hip joint (Fig. 4b), during approximately 25–75% of the stance phase, as opposed to extension moment in their study. Apart from these two differences, our results are almost identical to theirs. In our case, the EMG of the affected limb demonstrated an increase in electric activity for both the MH (Fig. 7a) and LH (Fig. 7b) from the mid-stance phase to the late-stance phase, and electric activity of the GL (Fig. 8b) increased from 50% of stance to the late-stance phase. It is important to note that there were differences in the background factors between their study and ours. Firstly, the cause of the disease differs: all four of their cases had neuropathy [7], whereas our case involved surgical resection. Secondly, three out of four of their cases usually used a cane or crutch [7]. Only one participant could walk unaided, but unlike our case, his knee was not hyperextended [7]. Moreover, his loss of strength occurred gradually, and he was stronger than the other three participants in lower extremity muscles not innervated by the femoral nerve [7]. The difference in walking style and muscle activity, especially in the hamstrings and gastrocnemius, may have contributed to the discrepancy between our results and theirs.

In our analysis, the hip extension moment was not increased or prolonged in the loading response. The plantar flexion absorption power of the affected ankle was increased in the late-stance phase and the muscle activities of the MH, LH. and GL were increased during 50–75% of the stance phase. Moreover, in the late-stance phase, the bilateral knees showed almost the same kinematic and kinetic results. During all phases, the kinetic results of the bilateral hips and ankles were almost the same. However, the hip flexion angle of the affected limb was smaller and the ankle plantar flexion angle of the affected limb was larger than that of the non-affected limb. This means that in the affected limb, the hip and ankle angles were adjusted in the all-stance phase to prevent knee collapse, and the MH, LH, and GL muscles contributed in the mid- and late-stance phases. The distal parts of MH and LH attach to the proximal tibia, while the proximal parts of GL and GM attach to the distal femur. It can be assumed that MH and LH make the proximal tibia posterior by extending the hip joint, and GL and GM make the distal femur posterior by dorsiflexing the ankle joint. Considering the above, our case is the first to use EMG to show that both the gastrocnemius and hamstrings need to work effectively to prevent knee collapse between the mid- and late-stance phases.

We have previously reported a case of gait analysis in a patient who had undergone quadriceps resection and patellectomy [3]. Similar to the current case, the previous report showed that the affected ankle maintained a plantar-flexed position throughout the stance phase. However, unlike the current case, the affected limb had a slight knee flexion position in all phases without initial contact and relatively high gastrocnemius activity (even in the loading response), and the MH was not so activated in the mid- and late-stance phase, and both the hip flexion and extension angles were equivalent to those of the non-affected limb. In the current case, the affected limb was hyperextended during 0–70% of the stance phase. The difference between our previous and current results may be explained by the hyperextension-induced locking mechanism of the knee, which could have suppressed the muscular activity of the gastrocnemius.

Considering these differences, in the previous report, the gastrocnemius may have contributed to maintaining a slight knee flexion at the initial contact or loading response. Moreover, the gastrocnemius activity for the affected limb was increased from approximately 80% to 100% of the stance phase in comparison with that in the current case, and the LH activity for the affected limb from 50% to almost 100% of the stance phase was equal to that measured in the current case. These results suggest that the gastrocnemius was more important in preventing knee collapse in the previous case than in the current case. When the affected knee remained slightly flexed during the stance phase, the ipsilateral hip extension did not require as much hamstring activity, and a regular gait could be maintained. Our previous case involved a child; therefore, the patient may have been able to gain a near-natural gait because he maintained slight flexion in his affected knee during almost the entire stance phase by properly controlling the ipsilateral hamstrings and gastrocnemius.

Therefore, adult patients who experience femoral nerve resection or injury may find it difficult to reacquire natural walking with the knee slightly flexed by controlling the hamstrings and gastrocnemius muscles.

To the best of our knowledge, this is the first report of gait analysis combined with electromyography for a patient who underwent femoral nerve resection. The patient was able to walk stably and unaided to prevent the affected knee from collapsing. We found that the hamstring and gastrocnemius of the affected limb worked together to keep the ipsilateral knee extended in the mid-stance phase and slightly flexed in the late-stance phase.

## Data Availability

The datasets used and/or analyzed during the current study are available from the corresponding author on reasonable request.

## Abbreviations

EMG: Electromyography
MRI: Magnetic resonance imaging
WI: Weighted image
VM: Vastus medialis
VL: Vastus lateralis
MH: Medial hamstrings
LH: Lateral hamstrings
GM: Medial heads of the gastrocnemius
GL: Lateral heads of the gastrocnemius
SENIAM: Surface EMG for the non-invasive assessment of muscles
MVC: Maximum voluntary contraction
